# Tannin-Based Microbicidal Coatings for Hospital Privacy Curtains

**DOI:** 10.3390/jfb14040187

**Published:** 2023-03-27

**Authors:** Petri Widsten, Satu Salo, Klaus Niemelä, Hanna Helin, Minna Salonen, Hanna-Leena Alakomi

**Affiliations:** 1 VTT Technical Research Centre of Finland Ltd., Tietotie 2, 02150 Espoo, Finland; 2 FIMLAB Laboratoriot Ltd., Arvo Ylpön katu 4, 33520 Tampere, Finland; 3 Hospital Nova of Central Finland, The Central Finland Health Care District Consortium, Hoitajantie 3, 40620 Jyväskylä, Finland

**Keywords:** antimicrobial, coating, curtain, *E. coli*, hospital, hygiene, MRSA, *S. aureus*, tannin

## Abstract

The goal of this study was to develop a sustainable, tannin-based option for silver-based and other current antimicrobial solutions for hospital privacy curtains. Commercial tree-derived tannins were characterized and their in vitro antibacterial properties against *Staphylococcus aureus* and *Escherichia coli* were determined. Hydrolysable tannins showed greater antibacterial efficacy than condensed tannins but differences in antibacterial efficacy between any of the tannins could not be attributed to their functional group content or molar mass. Outer membrane disruption was not a significant factor in antibacterial efficacy of tannins against *E. coli*. In a hospital field study, draw patches coated with hydrolysable tannins and affixed to privacy curtains reduced total bacteria count by 60% over eight weeks compared to their matching uncoated reference sides. In a follow-up laboratory study with *S. aureus*, very light spraying with water improved contact between bacteria and coating, enhancing the antibacterial effect by several orders of magnitude.

## 1. Introduction

The continuous spread of multidrug resistant organisms (MDROs) such as methicillin-resistant *S. aureus* (MRSA), enterococci, streptococci, and *Acinetobacter baumannii* in hospital environments is a serious problem that has caused millions of deaths due to untreatable post-operation wound infections and is threatening to undermine the future of medical treatments requiring surgical interventions. Standard hospital privacy curtains (HPCs) that are frequently touched by hospital staff and patients become rapidly colonised by microbes such as methicillin-resistant *S. aureus* and vancomycin-resistant *Enterococcus* (VRE) and are a well-established source of microbial transmission [1,2,3,4,5,6,7,8,9]. While good outcomes on pathogen reduction [10,11] have been reported for some types of antimicrobial fabrics and disinfectants used for HPCs, impregnation of HPCs with antimicrobial agents may fail to make a significant impact on pathogenic contamination HPCs [12], or the effects of disinfectant sprays are transient at best [12]. Moreover, regular cleaning of clinical surfaces such as HPCs according to standard cleaning practices with detergents and powerful disinfectants, such as improved hydrogen peroxide, fails to prevent bacteria from developing biofilms (bacteria embedded in exopolymeric substances) that periodically release bacteria back into the environment [11,13].

In an attempt to combat MDROs, many hospitals have switched from standard HPCs to disposable HPCs with integrated antimicrobial properties in which the polypropylene (PP), polycotton or other curtain fabric is embedded or coated with silver, quaternary ammonium chlorides (QACs) or combinations of materials. The antimicrobial efficacy of commercial HPCs is demonstrated in standard laboratory tests, such as the ISO 20743, in which the manufacturers typically report broad spectrum log 3 (99.9%) reductions against a variety of bacteria, viruses, and fungi. Published laboratory studies of HPC with QACs have also reported significant reductions in microbial burdens compared to the standard HPCs [14]. However, real-life testing of antimicrobial HPCs in hospital settings have shown variable results [10,12,15,16,17]. In one such investigation, silver integrated HPCs were as ineffective at reducing microbial burdens as were standard HPCs, whereas HPC with embedded QACs and polyorganosiloxane proved highly effective, extending the time of first contamination from the five weeks of standard curtains to >19 weeks [17]. In another study in a clinical environment, QAC-equipped HPCs reduced total microbial burden by 93% in reference to standard HPCs [10]. Further hospital studies of HPCs containing combinations of different antimicrobial elements have also produced results in favour of replacing standard HPCs with antimicrobial ones [12,15,16].

Trees are a treasure trove of compounds with proven antimicrobial properties [18], including tannins [19,20,21,22,23] that the trees produce to protect themselves from microbes, insects and larger herbivores. The main two types of tree tannins are hydrolysable tannins (HTs) and condensed tannins (CTs) [19,20,21,22,23,24,25,26].

**Figure 1:**
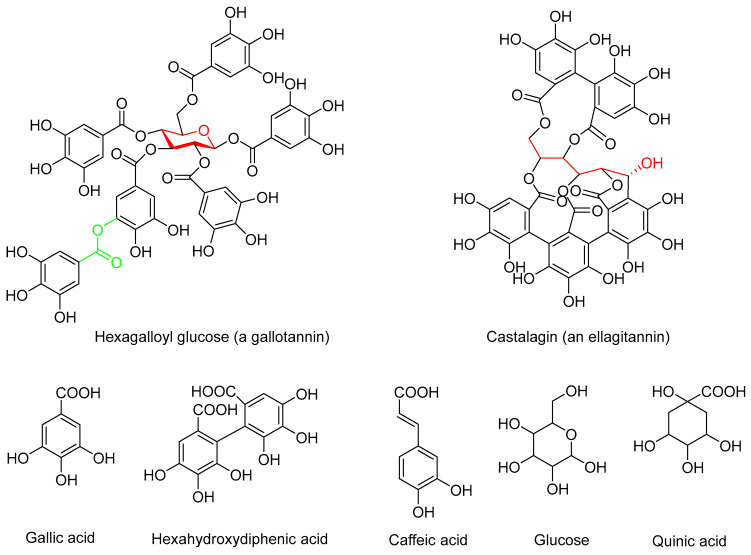
Examples of hydrolysable tannins (HTs) and their building blocks.

In the first instance of HT biosynthesis, gallotannins are formed via esterification of gallic acid and β-D-glucose, giving rise to mono- to pentagalloyl glucoses, which may add further galloyl units via formation of depside bonds (Figure 1). For example, in a commercial purified gallotannin (tannic acid) with a total phenol content (TPC) of ca. 900 mg/g catechin equivalents, the main constituents were hexa- to nonagalloyl glucoses [27]. Quinic acid is incorporated in the structure of some gallotannins in the same way as gallic acid [28], and tara tannins, for instance, contain galloyl- and caffeoylquinic acids [29,30]. The formation of ellagitannins involves oxidation of gallotannins followed by C-C coupling between their galloyl moieties, giving rise to hexahydroxydiphenic acid and its derivatives such as castalagin (Figure 1) [19,20,21,22]. Larger oligomeric and macrocyclic HTs can be built up via further esterification and coupling reactions that may involve both galloyl and hexahydroxydiphenoyl units.

The flavonoid biosynthetic pathway produces monomeric flavan-3-ols such as epicatechin and epigallocatechin that then form oligo- and polymeric proanthocyanidins, or CTs [19,23]. The individual flavonoid units of CTs are connected by C-C bonds between aromatic and aliphatic rings. Depending on the substituent pattern of the A- and B-rings, CTs are classified as procyanidins, prorobinetinidins, prodelphinidins, or profisetinidins.

HTs and CTs have proven antibacterial, antiviral, antifungal, and anti-biofilm activities that are largely linked to their ability to interact with proteins of extracellular microbial enzymes and those on the cell wall and membrane surfaces of microbes [19,20,21,22,23,24,25,26]. They may also act as siderophores, depriving microbes of iron, a crucial element for many of them. The exact mechanisms of antimicrobial action of individual HTs in particular tend to be elusive, due in part to the fact that HTs are typically poorly characterised and heterogeneous mixtures of different oligomers. In addition, configurational differences between otherwise closely related tannins, e.g., between epimers, can significantly influence their antimicrobial properties [22].

HTs and CTs are extracted in large quantities for leather tanning and other industries. They are obtained from the wood, bark, and other parts of several tree species, including mimosa and quebracho CTs and chestnut, valonea, and tara HTs. Bulk prices for these and cationised CTs typically range from 2 to 5 EUR/kg. Cationised CTs, applied as flocculants in wastewater treatment, are derivatives of CTs that contain quaternary amino groups [31]. Such tannins may have significant anti-biofilm activity, particularly against Gram-negative bacteria [26].

In accordance with the principles of a sustainable bioeconomy, the present investigation aimed at elucidating the mechanisms of action of tannins against nosocomial bacteria and the antibacterial efficacy of tannin coatings on hospital privacy curtains in laboratory and field conditions.

## 2. Materials and Methods

### 2.1. Tannins

Samples of commercial CT (mimosa and quebracho), cationic mimosa CT (Tanfloc SG and Tanfloc SH), HT (chestnut, valonea), and HT-rich tara pod powder containing ca. 40–60% tannin [32] were kindly provided free of charge by Christian Markmann GmbH, Hamburg, Germany. Spruce CT was prepared by soda cooking of spruce bark followed by acid precipitation (pH 2.5) of tannin from the spent liquor. Two extractions of tara pod powder were performed: (1) 20 g of powder was dispersed in 75% ethanol at a consistency of 10%, stirred at 1000 rpm and room temperature for 24 h, followed by filtering out the insoluble particles, evaporating the ethanol from the filtrate at reduced pressure, and freeze-drying the water phase (Tara-E tannin), and (2) 10 g of powder was extracted with Milli-Q water and filtered in the same way as the first extract and the filtrate then freeze-dried (Tara-W tannin).

### 2.2. Characterisation of Tannins

The hydroxyl and carboxyl contents of tannins were determined according to Granata and Argyropoulos [33] from freshly phosphitylated tannins by ^31^P NMR on a Bruker 500 MHz NMR spectrometer at room temperature using a previously published method. Nitrogen contents were determined as described by Nordlund [34]. Carbohydrates were determined by HPAEC-PAD from acid hydrolysed samples [35]. Molar mass measurements of tannins, dissolved in 0.1 M NaOH and filtered (0.45 μm), were performed by size exclusion chromatography using 0.1 M NaOH as the eluent (pH 13, 0.5 mL/min, T = 25 °C) on PSS MCX 1000 and 100,000 Å columns. The elution curves were detected using a Waters 2998 Photodiode Array detector at 280 nm. The weight (M_w_)- and number (M_n_)-average molar masses were calculated against polystyrene sulfonate standards (eight standards with a range of 3420−148,500 g/mol) using Waters Empower 3 (Milford, MA, USA) software.

### 2.3. Preparation and Function of Antimicrobial Curtain Coatings

Patches of commercial PP hospital privacy curtain (Elers Medical Finland Ltd., Helsinki, Finland) were cleaned with ethanol and corona-treated [36] to improve coating adhesion. The corona treatment involved passing a hand-held corona treater (BD-20AC Laboratory Corona Treater, Electro-Technic Products, Chicago, IL, USA) over the patch surface twice at a speed of ca. 2 m/min and at a distance of ca. 3 cm. Aqueous tannin solutions (Table 1) were then applied by brushing onto the corona-treated PP patches and viscose (Tringa^®^ by Paptic Ltd., Espoo, Finland) patches of similar size.

For the hospital field trial, eight PP patches and eight viscose patches coated with chestnut, valonea, tara-W/tara-E and TanFloc SG tannins (two coatings per tannin type and patch material) were prepared. Patches were attached at the end of existing curtains using stapler (see hospital field study). Staff and patients were instructed to grab the patches when drawing the curtains.

### 2.4. Antimicrobial Testing Methods

Antimicrobial efficacy of liquid samples (2.5% tannin dissolved in water) was tested using the modified 555 suspension test method [40]. The target microbes originated from VTT Culture Collection, including *S. aureus* VTT E-70045 and *E. coli* VTT E-94564. The principle of this method is to contact microbes with sample and measure the survival of microbes after the exposure time. Physiological salt water was used as a control. Tests were performed at room temperature. After a contact time of 24 h, survival of microbes was measured by culturing samples and incubating them (Plate Count Agar (PCA); 48 h, 37 °C). For antimicrobial surfaces the principle of the test was the same. One millilitre of inoculum was spread on the tested surface and covered with sterile plastic. After the contact time (24 h), microbes were diluted to 10 mL with peptone saline and cultured as above. Minimum inhibitory concentration (MIC) tests were performed according to a standard method [41]. Briefly, Mueller–Hinton (MH) broth was inoculated with colonies grown on agar and incubated at 37 °C overnight at a concentration of ca. 10^8^ cells/mL. The inoculum was diluted to a final concentration in the well of 5 × 10^5^ cfu/mL. The concentration of the inoculum was confirmed by plate count methods. Serial two-fold dilutions of the antimicrobial agents in four parallel wells were used to determine the MIC. The microwell plates were incubated at 37 °C for 24 h, and the MIC was determined as the lowest concentration of the antimicrobial agent to prevent growth.

Sampling of antimicrobial curtain patches in the hospital was performed by hospital personnel using COPAN M40 Transsystem swabs. In the sampling, the entire patch area was swabbed from side to side, up to down, and diagonally. The swabs were then placed in gel tubes and transported to a laboratory (FIMLAB, Tampere, Finland) for culturing in tryptic soy agar (TSA) plates within 24 h. The cultured swab samples were incubated for up to five days at two different temperatures (at first at 35 °C for two days then at room temperature for three days) after which the colonies were counted and identified. Identification was performed by Vitek MS (an automated mass spectrometry microbial identification system that uses Matrix Assisted Laser Desorption Ionization Time-of-Flight (MALDI-TOF, BioMerieux) technology). Identification at FIMLAB was performed using a clinical library with an identification reliability of 99.9%.

Laboratory testing in the follow-up study included a comparison of dry and moisture treatment methods on viscose-based curtain patches with and without (=control) tannin coating. Control patches and coated patches were contaminated with *S. aureus* by collecting colonies from a PCA plate and spreading them on surfaces with a non-woven cloth. Half of the control and coated samples were kept at room temperature as such while the other half were lightly wetted by spraying sterile water (Milli-Q filtrated) on the samples. After exposure times of 1 h, 3 h, 5 h, and 72 h, the bacteria were quantified by swabbing the surface with a cotton-tipped swab, diluting the bacteria in 5 mL peptone saline, and culturing them on PCA and incubating at 37 °C for 48 h.

The mechanisms of action of tannins against Gram-negative bacteria were studied using the 1-N-phenylnaphtylamine (NPN) uptake method of Alakomi et al. [42,43]. NPN is a hydrophobic probe whose quantum yield is greatly enhanced in a glycerophospholipid versus an aqueous environment. Uptake of NPN by bacterial membranes is manifested as fluorescence that indicates damage to the Gram-negative outer membrane, which normally can exclude hydrophobic substances.

The antibacterial activity of the tannins was studied at NPN concentrations of 0.25 mg/mL and 0.50 mg/mL using *E. coli* VTT E-94564 as the target microbe. EDTA was used as a control.

## 3. Results and Discussion

### 3.1. Characterisation of Tannins

**Figure 2:**
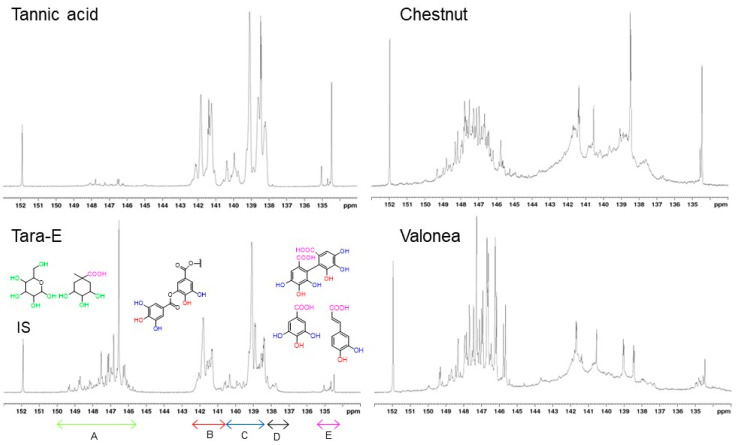
^31^P NMR spectra of hydrolysable tannins [44]. Peak assignments: (**A**) aliphatic hydroxyls (mostly in carbohydrate impurities), (**B**) *ortho*-disubstituted phenolic hydroxyls, (**C**) *ortho*-monosubstituted phenolic hydroxyls, (**D**) *ortho*-unsubstituted phenolic hydroxyls, (**E**) carboxylic acids. IS = internal standard. The spectrum of tara-W (not shown) was very similar to that of tara-E.

The HTs differ regarding their proportions of gallo- and ellagitannins as well as the molar masses of their constituent tannin oligo- and polymers, properties which may affect their antibacterial properties. The ratio of *ortho*-disubstituted and *ortho*-monosubstituted phenolic hydroxyls (PhOHds:PhOHms), integrated separately in ^31^P NMR spectra (Figure 2), can be used to assess whether a tannin is predominantly a gallo- or an ellagitannin. In pentagalloyl glucose, the prototypical gallotannin, each of the aliphatic hydroxyls of glucose is esterified with gallic acid but none of the galloyl units is further esterified with gallic acid via depside bonds. Its PhOHds:PhOHms is, thus, 0.5. As the number of depside bonds in the gallotannin molecule increases, so does PhOHds:PhOHms (e.g., 0.67 for decagalloyl glucose). Caffeoyl units (Figure 1) of tara gallotannins, however, contain only *ortho*-monosubstituted phenolic hydroxyls. In ellagitannins, PhOHds:PhOHms is higher than in gallotannins (e.g., 0.8 in castalagin, Figure 1). Although the total phenolic hydroxyl contents of the HT samples range from 5.08 to 14.30 mmol/g (Table 1), their PhOHds:PhOHms ratios are remarkably similar at ca. 0.5 except for valonea tannin (0.77) that apparently has a high ellagitannin content. It is difficult to estimate the proportion of ellagitannin in tara-E and tara-W tannins due to the occurrence unknown quantities of caffeoyl units.

The most prominent components of tannic acid (a commercial purified gallotannin) analysed in another investigation included penta- to nonagalloyl glucoses and digallic acid [27].

Galloyl units (and caffeoyl units of tara tannins) of gallotannins contain mostly *ortho*-monosubstituted phenolic hydroxyls and only the central phenolic hydroxyl of galloyl units is *ortho*-disubstituted, while two out of three phenolic hydroxyls of hexahydroxydiphenoyl units of ellagitannins (Figure 1) are *ortho*-disubstituted (Figure 2, Table 2) ranging from the 0.33 of a pure gallotannin (or less with caffeoyl units present) to the 2 of a pure ellagitannin. Based on the ratios calculated, all the HTs are mixtures of gallo- and ellagitannins with the gallotannins dominating and their proportion in the HTs increasing in the order valonea < chestnut < tara-W < tara-E.

Most of the phenolic hydroxyls of tara tannin were of *ortho*-monosubstituted type, consistent with the presence of mostly galloyl- and caffeoyl units of gallotannins rather than hexahydroxydiphenoyl units of ellagitannins. Only a minor number of aliphatic hydroxyls associated with the central glucose/quinic acid units of gallotannins were present, indicating a high degree of esterification and that nearly all the aliphatic hydroxyls occurred in carbohydrate impurities.

### 3.2. Antibacterial Efficacy of Tannin Powders

HTs and CTs of different types (Table 1) considered for coating application were first screened by testing them directly as powders (Table 3). A bactericidal effect (≥log 3 reduction) against both *S. aureus* and *Escherichia coli* (*E. coli*) in the modified 555 suspension test, achieved with most of them (Figure 1), was the selection criterion for coating trials. Excluded were, thus, the non-cationised CTs that failed to produce a bactericidal effect against both species of bacteria. In general, there is no obvious correlation between the antibacterial properties and the characteristics of the tannins in Table 2. This agrees with the recent findings of Villanueva et al. [26] who found the antibacterial activity of tannins to be only weakly correlated with their content of phenolic hydroxyl groups.

### 3.3. Mechanisms of Action of Tannins

**Figure 3:**
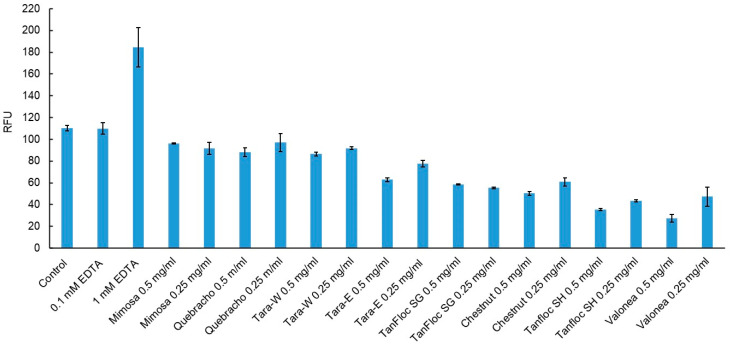
NPN uptake of *E. coli* cells caused by tannins quantified by fluorescence (relative fluorescence units—RFU).

The classical chelator EDTA significantly destabilized the outer membranes of *E. coli* as indicated by an increased uptake of NPN fluorochrome, whereas the tannins investigated showed minor efficacy in terms of weakening the outer membrane of *E. coli*, indicating a different mode of action (Figure 3). A decrease in NPN uptake in tannin samples compared to the control cells indicates interaction with other parts of cell membranes. It has been suggested [25] that tannins bind to the charged groups of membrane proteins and introduce changes into their functions. Several other mechanisms have been proposed to have a role in the antibacterial activity of hydrolysable tannins containing free galloyl groups [24].

### 3.4. Laboratory Testing of Tannin Coatings before Hospital Field Study

**Figure 4:**
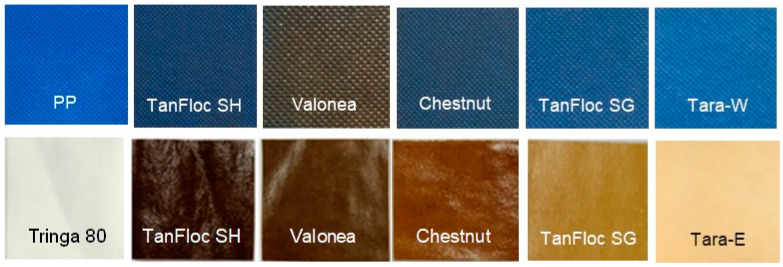
Uncoated and tannin-coated PP (top row) and Tringa 80 viscose (bottom row) privacy curtains.

**Figure 5:**
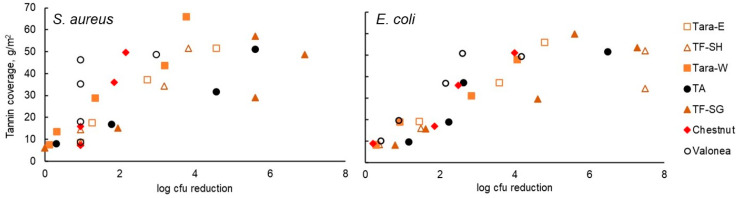
Antibacterial efficacy of tannin coatings on PP privacy curtains against *S. aureus* and *E. coli.*

**Figure 6:**
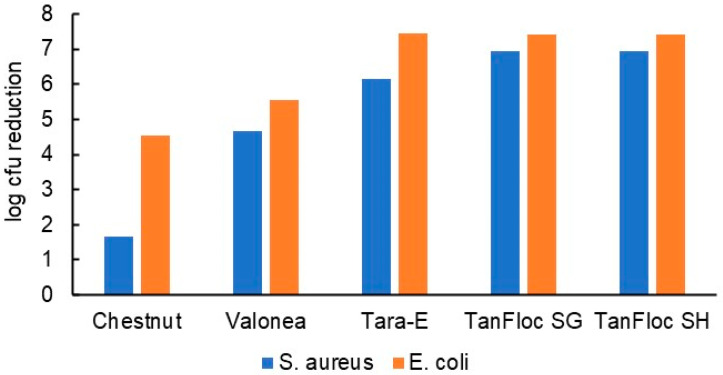
Antibacterial efficacy of tannin coatings on viscose (ca. 50 g/m^2^ tannin).

Tannin coatings of up to 65 g/m^2^ on 5 × 5 cm PP pieces (Figure 4) were tested against *S. aureus* and *E. coli*. The antibacterial effect tended to increase with increasing tannin coverage (Figure 5), which is not surprising given that the test method allowed for the tannins to dissolve in the test medium. The inhibitory effect observed for each tannin was quite linearly dose-dependent, the cationised tannins being somewhat more effective in reaching the highest degrees of inhibition with lower doses. Some of the tannins were also coated onto viscose at ca 50 g/m^2^ to ascertain whether good coating adhesion and antibacterial efficacy could be obtained with viscose as the base material. Indeed, the results confirm this to be the case (Figure 6). Based on these results, all tannins (except for the TA reference) were taken forward to the hospital field trial.

### 3.5. Hospital Field Study

**Figure 7:**
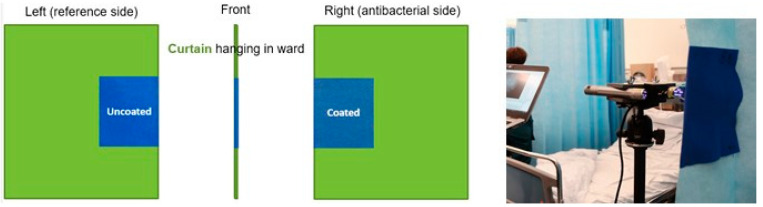
Installation of draw patches with a tannin-coated and uncoated side on hospital curtains [44].

In the hospital field trial, 16 PP and viscose curtain patches (Figure 4) were affixed on privacy curtains in different hospital wards around their edge in such a way (Figure 7) that anyone grabbing one to move the curtain would forcibly be touching both the uncoated (reference) and tannin-coated sides.

**Figure 8:**
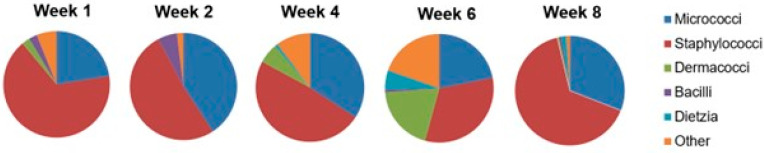
Distribution of bacterial genera detected on privacy curtain patches from week 1 to week 8 of the hospital field trial. Bacteria on both sides of all the patches are included [44].

**Figure 9:**
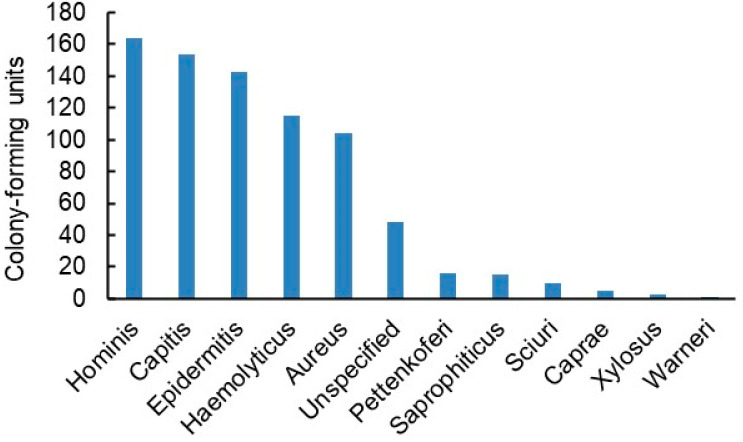
Species distribution of *Staphylococci* found on curtain patches from week 1 to week 8. Numbers of identified isolates. Bacteria originating from both sides of all the patches are included [44].

Staff and patients were advised to only use the patches for drawing the curtains. They were in place for eight weeks, during which 17 different bacterial genera were identified on them (Figure 8). While the proportions of the genera changed from week to week, the predominant ones by far were *Staphylococci* and *Micrococci*. Each major genus was represented by several species—13 alone in the case of *Staphylococci* (Figure 9). Overall, a vast majority of the bacteria were typical human skin bacteria such as *S. hominis* while the incidence of pathogenic bacteria such as *S. aureus* was low.

**Figure 10:**
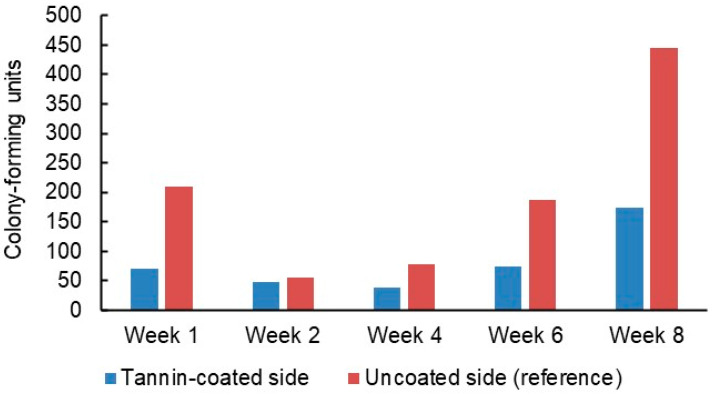
Total number of viable bacteria detected on PP and viscose privacy curtain patches during an 8-week hospital field trial.

**Figure 11:**
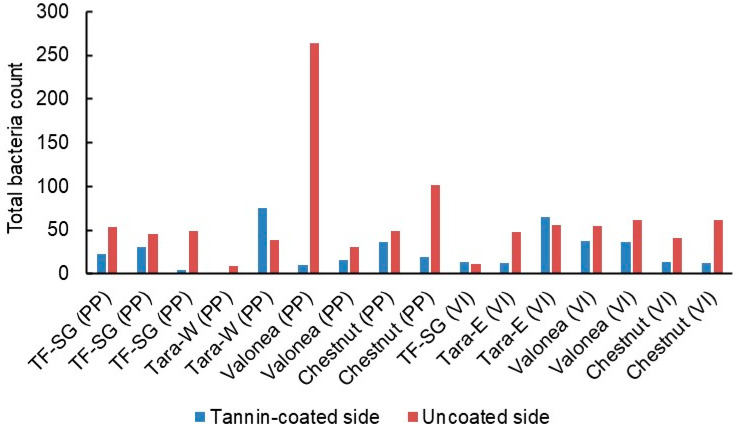
Total number of bacteria found on PP and viscose (VI) curtain patches during weeks 1–8 of the hospital field trial (1–3 replicates per tannin).

The number of bacteria found on the patches decreased after the first week but then climbed steadily during the rest of the trial period (Figure 10). Every week, fewer bacteria were detected on the tannin-coated sides than on the uncoated reference sides. Due to the relatively small number of patches, it was not possible to compare the performance of different types of tannin or patch material (PP or viscose, Figure 11) in a statistically meaningful way. On average, there were 60% fewer bacteria on the tannin-coated sides compared to the reference sides and there were fewer bacteria on the tannin-coated side than on the corresponding reference side for 13 of the 16 patches.

### 3.6. Follow-Up Study

**Figure 12:**
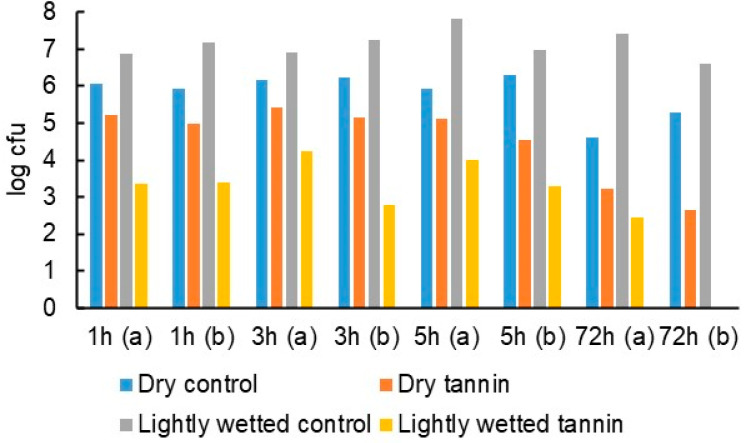
Effect of light wetting on antibacterial efficacy of tara tannin coating on cellulose sheet (a and b denote duplicate samples).

While the above results were positive considering that they were achieved at typically dry conditions under which only limited contact occurs between bacteria and tannin coatings, it was hypothesised that substantially larger reductions in bacteria counts could be obtained if the contact and interaction between bacteria and tannin were to be improved by light wetting of bacteria-bearing patch surfaces. The hypothesis was put to the test at laboratory conditions by depositing *S. aureus* bacteria directly from colonies growing on agar on PP patches coated with tara tannin or left uncoated. The patches were either left to incubate for 72 h without further action or lightly wetted with a fine spray of sterile water at the beginning of the incubation period. As expected, the wetting increased the bacteria count on the uncoated surfaces (Figure 12). There was a modest decline in bacteria on the dry tannin-coated surfaces—a result that is in line with those of the hospital field study. However, the effect of wetting drastically improved the antibacterial efficacy of the tannin-coated sides so that at the end of the 72-h incubation time they had achieved a bactericidal effect compared to the dry uncoated surfaces, thus validating the hypothesis. The practical implication of this result is that periodic light wetting with water could be part of the regular hygiene protocol of hospital privacy curtains to maintain their antimicrobial efficacy at a high level.

## 4. Conclusions

In traditional suspension tests of antibacterial efficacy, hydrolysable tannins outperformed condensed tannins, but differences in antibacterial efficacy between any of the tannins could not be attributed to their functional group content or molar mass. Disruption of outer membranes of Gram-negative bacteria (*E. coli*) was not found to be a significant factor in antibacterial efficacy of tannins. In a hospital study, coating draw patches of privacy curtains with tannin reduced their bacterial loading by 60%. In a follow-up laboratory investigation, it was found that a light wetting that could be incorporated into regular hospital hygiene practices may boost this antibacterial efficacy by orders of magnitude.

## Figures and Tables

**Table 1:** Tannins used for privacy curtain coatings.

Species of Origin	Tree Component	Tannin Type	Main Tannin Subtype
Valonea oak (*Quercus ithaburensis*)	Acorn cups	HT ^1^	Gallo- and ellagitannins [37,38]
Tara (*Caesalpina spinosa*)	Seed pods	HT ^1^	Gallotannins including galloquinic and caffeoylquinic acid units) [30,32,39]
Chestnut (*Castanea* sp.)	Bark/wood	HT ^1^	Gallo- and ellagitannins [28,39]
Mimosa (*Acacia mearnsii*)	Bark	CT ^1^	Prorobinetinidin [27,37]; catechin and gallocatechin di- and oligomers [27]
Quebracho (*Schinopsis* sp.)	Bark	CT ^1^	Profisetinidin [37]
Mimosa (*Acacia mearnsii*)	Bark	Cationic CT ^1^	Cationised [31] mimosa tannin

^1^ Samples of commercial tannins (with bulk prices ranging from 1.9 to 3.9 EUR/kg) were kindly provided free of charge by Christian Markmann GmbH, Hamburg, Germany.

**Table 2:** Characterisation of commercial tannins.

Tannin	Aliphatic OH, mmol/g		Phenolic OH, mmol/g				COOH, mmol/g	N, %	Carbohydrates, %	Molar Mass, g/mol	
	In Tannin	Total	*Ortho*-Substitution			Total				M_w_	M_n_
			None	Mono	Di						
Tannic acid	0.02	0.44	0.00	9.56	4.71	14.30	0.56	0.01	1.4	883	732
Chestnut	0.45	4.55	0.20	4.06	2.26	7.52	0.36	0.11	12.6	1456	951
Valonea	0.51	5.15	0.10	2.39	1.83	5.08	0.40	0.44	9.1	1269	869
Tara-E	0.16	4.68	0.04	7.14	3.62	10.84	0.25	0.17	5.2	925	725
Tara-W	0.19	4.97	0.02	6.37	3.25	9.66	0.25	0.42	7.1	913	718
Tara powder								1.17			
Mimosa		5.30				8.52	0.32	0.16	6.7	1697	1073
Quebracho		3.78				7.69	0.42	0.57	8.7	1820	1161
TanFloc SG								7.60	4.2	2677	1197
TanFloc SH								6.61	3.1	3178	1273

**Table 3:** Antibacterial efficacy of tannin powders. Upper two result rows: reduction in log cfu/mL in modified 555 suspension test with 25 mg/mL tannin solution. Lower two result rows: MIC test (minimum concentration of tannin inhibiting bacterial growth).

Tannin Type			Hydrolysable				Condensed		Cationised Condensed ^2^	
Tannin	Ampicillin (Control)	Chestnut	Tara ^1^	Tara-E	Tara-W	Valonea	Mimosa	Quebracho	TF-SG	TF-SH
*S. aureus* ^3^ log reduction		3.8	4.2	5.0	2.1	3.6	3.1	0.5	5.0	5.0
*E. coli* ^4^ log reduction		5.0	5.0	5.0	5.0	5.0	2.1	3.0	5.0	5.0
*S. aureus* ^3^	0.25	0.83	5.7	8.0	8.0	0.58	0.58	1.67	1.44	>8.0
MIC, mg/mL										
*MRSA* ^5^										
MIC, mg/mL	2.00	1.0	8.0	4.0	1.0	0.5	1.0	2.0	0.5	>8.0

^1^ Tara pod powder as received (ca. 53% tannin); ^2^ TanFloc SG and TanFloc SH: cationised mimosa tannins; ^3^ *S. aureus* VTT E-70045; ^4^ *E. coli* VTT E-94564; ^5^ *MRSA* VTT E-183582.

## Data Availability

Data will be made available on request.
